# Enhanced *AZIN1* RNA editing and overexpression of its regulatory enzyme ADAR1 are important prognostic biomarkers in gastric cancer

**DOI:** 10.1186/s12967-018-1740-z

**Published:** 2018-12-18

**Authors:** Yoshinaga Okugawa, Yuji Toiyama, Kunitoshi Shigeyasu, Akira Yamamoto, Tsunehiko Shigemori, Chengzeng Yin, Takashi Ichikawa, Hiromi Yasuda, Hiroyuki Fujikawa, Shigeyuki Yoshiyama, Junichiro Hiro, Masaki Ohi, Toshimitsu Araki, Masato Kusunoki, Ajay Goel

**Affiliations:** 1 Department of Gastrointestinal and Pediatric Surgery, Division of Reparative Medicine, Institute of Life Sciences, Mie University Graduate School of Medicine, Tsu, Mie Japan; 2 Department of Gastroenterological Surgery, Okayama University Graduate School of Medicine, Dentistry, and Pharmaceutical Sciences, Okayama, Okayama Japan; 3 Center for Gastrointestinal Research and Center for Translational Genomics and Oncology, Baylor Scott & White Research Institute and Charles A. Sammons Cancer Center, Baylor University Medical Center, 3410 Worth Street, Suite 610, Dallas, TX 75246 USA

**Keywords:** *AZIN1*, RNA editing, Gastric cancer, Lymph node metastasis, Peritoneal metastasis, Prognosis

## Abstract

**Background:**

Adenosine-to-inosine (A-to-I) RNA editing is catalyzed by adenosine deaminases acting on RNA (ADAR) enzymes. Recent evidence suggests that RNA editing of antizyme inhibitor 1 (*AZIN1*) RNA is emerging as a key epigenetic alteration underlying cancer pathogenesis.

**Methods:**

We evaluated *AZIN1* RNA editing levels, and the expression of its regulator, ADAR1, in 280 gastric tissues from 140 patients, using a RNA editing site-specific quantitative polymerase chain reaction assays. We also analyzed the clinical significance of these results as disease biomarkers in gastric cancer (GC) patients.

**Results:**

Both *AZIN1* RNA editing levels and ADAR1 expression were significantly elevated in GC tissues compared with matched normal mucosa (P < 0.0001, 0.0008, respectively); and *AZIN1* RNA editing was positively correlated with ADAR1 expression. Elevated expression of ADAR1 significantly correlated with poor overall survival (P = 0.034), while hyper-edited *AZIN1* emerged as an independent prognostic factor for OS and disease-free survival in GC patients [odds ratio (OR):1.98, 95% CI 1.17–3.35, P = 0.011, OR: 4.55, 95% CI 2.12–9.78, P = 0.0001, respectively]. Increased *AZIN1* RNA editing and ADAR1 over-expression were significantly correlated with key clinicopathological factors, such as advanced T stage, presence of lymph node metastasis, distant metastasis, and higher TNM stages in GC patients. Logistic regression analysis revealed that hyper-editing status of *AZIN1* RNA was an independent risk factor for lymph node metastasis in GC patients [hazard ratio (HR):3.03, 95% CI 1.19–7.71, P = 0.02]. Conclusions: *AZIN1* RNA editing levels may be an important prognostic biomarker in GC patients, and may serve as a key clinical decision-making tool for determining preoperative treatment strategies in GC patients.

**Electronic supplementary material:**

The online version of this article (10.1186/s12967-018-1740-z) contains supplementary material, which is available to authorized users.

## Background

RNA editing refers to the post-transcriptional epigenetic modification of RNA sequences through insertion, deletion, or nucleotide conversion, resulting in an increased diversity of the transcriptomic repertoire. Adenosine-to-inosine (A-to-I) RNA editing is primarily mediated by adenosine deaminase acting on RNA (ADAR) enzymes, and is the predominant form of RNA editing in humans [1]. It involves the conversion of adenosine to inosine, which acts in a similar manner to guanosine in the general cellular machinery [2]. However, RNA editing in the coding region of mRNA molecules change the amino acid sequence of the encoded protein, with subsequent negative impact on the functionality of the corresponding protein [3]. Although genome-wide A-to-I editing was initially thought to be a rare event which was limited to coding exons, advances in next-generation sequencing technologies and bioinformatic tools have now allowed revealed identification of hundreds of thousands of RNA editing sites throughout the human transcriptome. Not surprisingly, RNA editing is gaining attention in the field of cancer research, and emerging evidence suggests that A-to-I RNA editing permits transcript localization and degradation via protein recoding, alternative splicing, and microRNA regulation; thus facilitating cancer evolution and pathogenesis [4, 5]. Furthermore, the selective distribution of RNA editing loci and their biological roles indicate the potential for clinically relevant diagnostic and prognostic tools capable of accurately assessing aberrant RNA editing involved in cancer progression and therapeutic resistance.

Chen and colleagues performed the first systematic and comprehensive analysis using a sequencing-based approach [6], and demonstrated that *AZIN1* RNA was specifically enhanced in HCC tissues, and significantly correlated with disease progression in HCC patients.* AZIN1* belongs to the antizyme inhibitor family, and plays a role in maintaining polyamine homeostasis which are important for various cellular functions, including cell growth [7, 8]. This hypothesis was supported by the findings that neutralization of a key inhibitor of the polymerase synthesis pathway through *AZIN1* RNA editing permitted unimpeded tumor growth and proliferation [6]. Recently, our group also revealed that *AZIN1* confers a gain-of-function phenotype frequently through A-to-I conversions via ADAR1, which can promote ornithine decarboxylase (ODC) and polyamines accumulation—conditions that are associated with aggressive tumors [6, 9].

The incidence of gastric cancer (GC) in developed countries has fallen significantly, however, this malignancy remains the fourth most common cancer and the second leading cause of cancer-related deaths worldwide [10]. Approximately, one third of GC patients are first diagnosed at late stages with a locally-advanced or metastatic disease. This highlights the need for identification and development of robust biomarkers that can allow early detection, as well as predict postoperative tumor recurrence, to improve the overall morbidity and mortality associated with gastric neoplasia.

Currently, several well-known antigens, including carcinoembryonic antigen (CEA), cancer antigen 19-9 (CA19-9) and cancer antigen 72-4 (CA72-4), or serological biopsy using Pepsinogen I and II have been investigated in the context of GC [11, 12]. Although various targets have been suggested to serve as potential biomarkers in patients with GC, biomarkers with adequate sensitivity and specificity for implementation in GC screening and risk stratification remain unavailable, but represent an active area of research.

Work from our group and others have previously identified several epigenetic alterations that could serve as biomarkers for diagnosis, prognosis, and metastasis prediction in patients with various gastrointestinal cancers [13–16]. More recently, we have also revealed the role of altered RNA editing levels and its functional consequence in colorectal cancer [17]. In the current study, we for the first time, investigate the RNA editing status of the antizyme inhibitor 1 gene (*AZIN1*) and the expression pattern of its regulatory protein, ADAR1, in the primary tumor tissues and matched normal mucosa from patients with gastric cancer, with an emphasis to gain insights into the clinical significance of these events in this malignancy.

## Materials and methods

### Patients and specimen collection

This study included analysis if 280 gastric tissues obtained from 140 patients (110 men and 30 women) who underwent surgery for GC at the Mie University Hospital, Japan, from 2000 to 2009. The study included patients from whom matched tumor and normal mucosa tissues were available for analysis. The mean patient age was 66.8 years (range 18–90 years). No patient received chemotherapy or radiotherapy prior to surgery and no perioperative mortality was observed. The diagnosis of GC was confirmed in all 140 patients based on clinicopathological findings. All patients were classified according to the Japanese Classification of Gastric Carcinoma [18]: 23 patients had stage I disease, 39 had stage II, 39 had stage III, and 39 had stage IV. Distal or total gastrectomy with D2 lymphadenectomy was performed in patients who underwent curative resection. Patients with liver, peritoneal, or distant metastasis underwent palliative gastrectomy with D1 lymphadenectomy. The mean follow-up was 26.5 months (range 1–79 months). Sixty-four patients died from cancer-related causes during the study period. All tissue specimens were immediately preserved in *RNAlater* following surgical resection (Qiagen, Chatsworth, CA, USA) and stored at − 80 °C until RNA extraction. Written informed consent was obtained from each patient, and the study was approved by the institutional review boards of all institutions.

### Total RNA extraction and cDNA synthesis

*RNAlater*-preserved surgical specimens were homogenized with a Mixer Mill MM300 homogenizer (Qiagen). Total RNA from tissues was isolated using RNeasy Mini kits (Qiagen) according to the manufacturer’s instructions. cDNA was synthesized from 1.0 µg total RNA using an Advantage RT PCR-kit (Clontech Laboratories Inc., Mountain View, CA, USA).

### RNA editing site-specific quantitative PCR

RNA editing of *AZIN1* was analyzed by RNA editing site-specific quantitative PCR developed by Crews and colleagues [19]. In brief, specific primers for wild-type and edited *AZIN1* (Chr:8, Position:103841636, Region:Exon) [6, 20, 21] were established. The *AZIN1* edited/wild-type ratio was calculated based on the differences in Ct values in a SYBR Green-based real-time PCR assay, using the formula 2-(Ct edit − Ct wild-type). Primer sequences for these reactions are shown in Additional file 1: Table S1).

### Real-time quantitative PCR

Quantitative reverse transcription PCR analysis was performed using the StepOne Real Time PCR System (Applied Biosystems, Foster City, CA, USA). ADAR1 and glyceraldehyde 3-phosphate dehydrogenase (GAPDH) mRNA expression levels were measured using Power SYBR Green Master Mix (Applied Biosystems). Sequence information on these primers is provided in Additional file 1: Table S1. We performed 40 cycles of amplification under the following conditions: denaturation at 95 °C for 10 s, annealing at 60 °C for 10 s, and elongation at 72 °C for 20 s. After amplification, the products were subjected to a temperature gradient ranging from 68 °C to 95 °C at 0.2 °C/s under continuous fluorescence monitoring to produce a melting curve of the products. After proportional background adjustment, the fit-point method was used to determine the cycle in which the log-linear signal was distinguished from the background, and that cycle number was used as a crossing-point value. Expression levels of target and GAPDH transcripts were evaluated using Applied Biosystems StepOne Software v2.1. Relative expression of each mRNA was determined using the ΔΔCt method, as described previously [15, 16].

### Statistical analysis

Results were expressed as mean ± standard deviation, and all statistical analyses were performed using Medcalc version 16.4.3 (Broekstraat 52, 9030; Mariakerke, Belgium). Differences between groups were analyzed by χ^2^ text, Mann-Whitney test, and one-way ANOVA, as appropriate. F-tests or Levene tests were used to assess the equality of variance for comparable groups. For time-to-event analyses, survival estimates were calculated by Kaplan–Meier analysis and groups were compared using log-rank tests. Receiver operating characteristic curves were established to determine the cut-off values for analyzing each aim of prognosis by Youden’s index. Overall survival was measured from the date the patient underwent surgery until the date of death resulting from any cause, or until the last known follow-up in patients that were still alive. In the entire GC population cohort we analyzed, the highest sensitivity and specificity thresholds for determining OS were found using a peak *AZIN1* RNA editing levels and ADAR1 expression cut-off point of 0.057 and 0.062, respectively. Disease-free survival was measured from the date the patient underwent curative surgery to the date of disease recurrence, death from any cause (i.e., cancer-unrelated deaths were not censored), or until last contact with the patient. In the curatively resected GC population, the highest sensitivity and specificity for DFS was determined using a peak *AZIN1* RNA editing levels and ADAR1 expression cut-off point of 0.096 and 0.062, respectively. Cox proportional hazards models were used to estimate hazard ratios for death or recurrence. Assumption of proportionality was confirmed for Cox proportional hazards analyses by generating Kaplan–Meier survival curves (e.g., high vs. low expression groups) and by ensuring that the two curves did not intersect. Multivariate logistic regression models were used to predict factors influencing lymph node metastasis. Forced-entry regression was used to include these variables in all multivariate equations to determine if any of the predictors affected the outcome after adjusting for known confounders. All P values were two-sided, and P < 0.05 was considered statistically significant.

## Results

### Dysregulated pattern of* AZIN1* RNA editing and over-expression of ADAR1 in gastric cancer

**Fig. 1:**
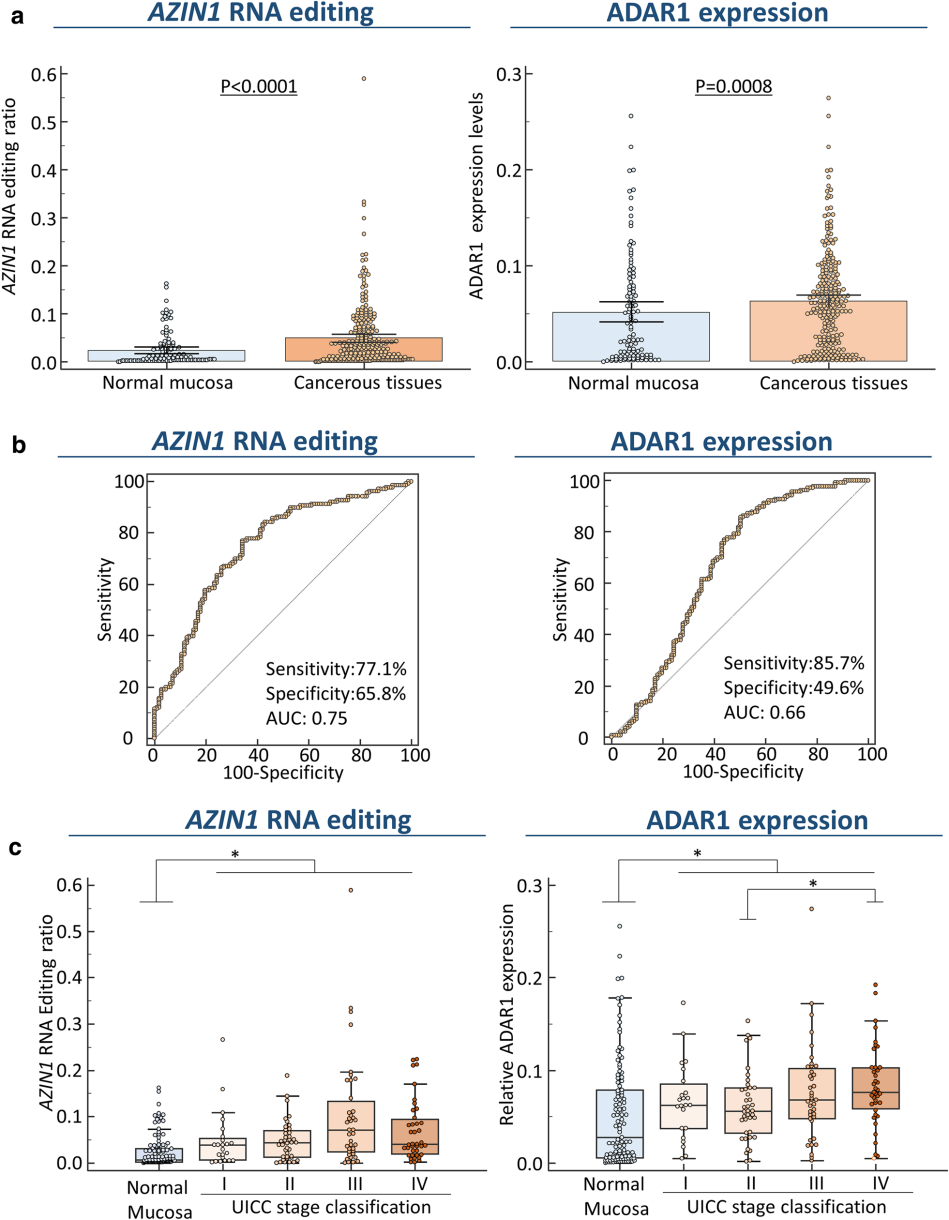
Dysregulation of* AZIN1* RNA editing and ADAR1 expression status in tissues from GC patients. **a*** AZIN1* RNA editing levels (left) and ADAR1 expression levels (right) were significantly increased in primary cancer tissues compared with adjacent normal mucosa. **b** ROC curve analysis for* AZIN1* RNA editing levels (left) and ADAR1 expression levels (right) for distinguishing between GC and normal gastric mucosa.* AZIN1* RNA editing levels and ADAR1 expression levels could distinguish GC tissues from normal gastric mucosa with area under the curve values of 0.75 (95% CI 0.69–0.8), 0.66 (95% CI 0.6–0.84), respectively. **c** Scattergrams of* AZIN1* RNA editing levels (left) and ADAR1 expression levels (right) according to UICC classification in gastric cancer patients

We first analyzed *AZIN1* RNA editing levels and expression of its regulatory enzyme ADAR1 to assess their expression patterns in GC tissues. Profiling analysis revealed that *AZIN1* RNA editing levels were significantly elevated in GC tissues compared with matched normal mucosa (P < 0.0001, Fig. 1a). Furthermore, ADAR1 was also significantly over-expressed in gastric cancer tissues compared with adjacent matched normal mucosa (P = 0.0008, Fig. 1a). Receiver operating characteristic (ROC) curves were used to evaluate the sensitivity and specificity of each dysregulated molecule for distinguishing GC from normal tissues. Notably, *AZIN1* RNA editing and ADAR1 expression displayed considerable diagnostic accuracy, with area under the curve values of 0.75 (95% confidence interval (CI) 0.69–0.8) and 0.66 (95% CI 0.6–0.72), respectively (Fig. 1b). Furthermore, we evaluated the relationship between *AZIN1* RNA editing levels and ADAR1 expression in GC patients, and showed that *AZIN1* RNA editing was positively correlated with ADAR1 expression in this cohort (P < 0.0001, r = 0.45).

### Correlation of* AZIN1* RNA editing levels and ADAR1 over-expression with disease progression in GC patients

We next evaluated the correlations between *AZIN1* RNA editing levels and ADAR1 expression with various clinicopathological factors to determine the clinical significance of our findings in GC patients (Table 1). Increased levels of *AZIN1* RNA editing were significantly correlated with established clinicopathological factors such as advanced T stage (P = 0.031), presence of lymph node metastasis (LNM; P = 0.021), and higher TNM stages (P = 0.028) in GC patients. Furthermore, over-expression of ADAR1 was also significantly associated with presence of LNM (P = 0.029), distant metastasis (P = 0.006), and more advanced TNM stages (P = 0.004) in these patients (Table 1, Fig. 1c).

**Table 1:** Correlation between clinicopathological Variables and *AZIN1* RNA editing and ADAR1 expression in Gastric Cancer patients

Variable	n	*AZIN1* RNA editing		*P value*	ADAR1 expression		*P value*
		High (n = 56)	Low (n = 84)		High (n = 78)	Low (n = 62)	
Gender							
Male	110	44	66	1	62	48	0.77
Female	30	12	18		16	14	
Age (year)							
69≥^a^	72	31	41	0.45	42	30	0.52
>69	68	25	43		36	32	
Histological type							
Intestinal type	69	29	40	0.63	41	28	0.39
Diffuse type	71	27	44		37	34	
Pathological T category							
pT1/2	50	14	36	0.031*	24	26	0.17
pT3/4	90	42	48		54	36	
Vessel invasion							
Present	111	43	68	0.55	62	49	0.95
Absent	29	13	16		16	13	
Lymphovascular invasion							
Present	128	52	76	0.62	74	54	0.1
Absent	12	4	8		4	8	
Lymph node metastasis							
N0	43	11	32	0.021*	18	25	0.029*
N1	97	45	52		60	37	
Peritoneal metastasis							
P0	117	43	74	0.08	61	56	0.06
P1	23	13	10		17	6	
Distant metastasis							
M0	101	39	62	0.59	49	52	0.006*
M1	39	17	22		29	10	
UICC TNM classification							
Stage I	23	5	18	0.028*	11	12	0.004*
Stage II	39	12	27		15	24	
Stage III	39	22	17		23	16	
Stage IV	39	17	22		29	10	

* *P *< 0.05

^a^The median age at surgery is 69 years in this cohort

### Associations between* AZIN1* RNA editing with tumor recurrence and survival outcomes in GC patients

**Fig. 2:**
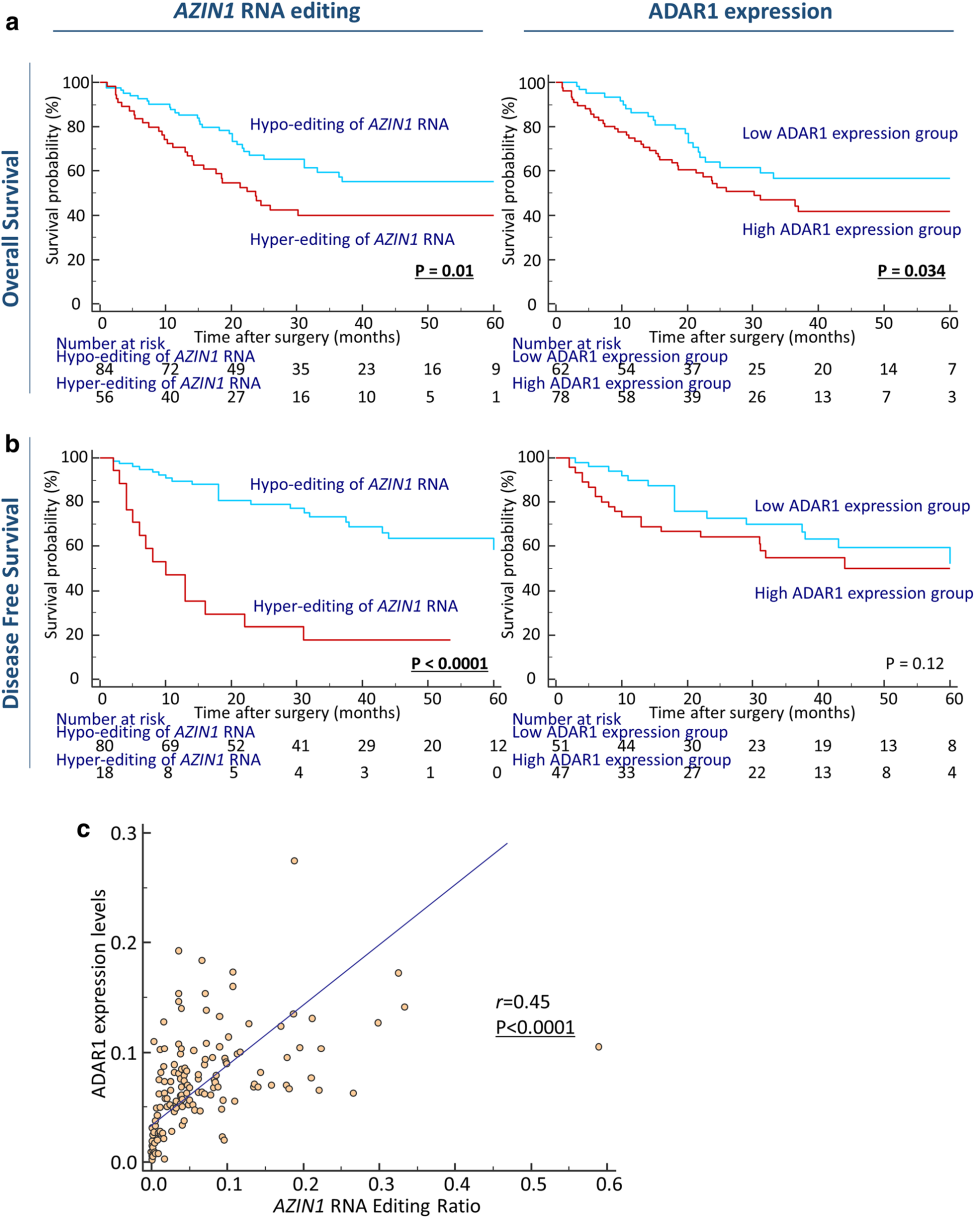
Associations between* AZIN1* RNA editing with tumor recurrence and survival outcomes in GC patients. **a** OS in GC patients in relation to* AZIN1* RNA editing (left) and ADAR1 expression levels (right). OS was significantly lower in GC patients with high* AZIN1* RNA editing levels or high ADAR1 expression levels compared with those with low levels of either parameter (*AZIN1* RNA editing; P = 0.01, ADAR1 expression; P = 0.034, log-rank test). **b** Kaplan–Meier survival curves for DFS in GC patients in relation to* AZIN1* RNA editing (left) and ADAR1 expression levels (right). DFS was significantly lower in GC patients with high* AZIN1* RNA editing levels or high ADAR1 expression levels compared with those with low levels of either parameter. **c** Correlation between ADAR1 expression and* AZIN1* editing in gastric cancer patients. (*AZIN1* RNA editing; P < 0.0001, ADAR1 expression; P = 0.12, log-rank test). All statistical tests were two-sided

We performed time-to-event analyses for the evaluation of the prognostic impact of *AZIN1* RNA editing status and ADAR1 expression in terms of overall survival (OS) and disease-free survival (DFS) in GC patients. We determined survival cut-off thresholds for each candidate derived from the ROC curve analyses with Youden’s index. Interestingly, both *AZIN1* RNA hyper-editing status and overexpression of ADAR1 were significantly correlated with poor OS (OS: *AZIN1* RNA editing; P = 0.01, ADAR1 expression; P = 0.034, log rank test; Fig. 2a). Interestingly, only increased *AZIN1* RNA status was significantly correlated with poor DFS (*AZIN1* RNA editing; P < 0.0001, ADAR1 expression; P = 0.12, log rank test; Fig. 2b). We carried out multivariate Cox regression analysis to determine the clinical significance of *AZIN1* RNA editing status as a prognostic biomarker of recurrence and prognosis in GC patients. Enhanced levels of *AZIN1* RNA editing emerged as an independent prognostic factor for OS in GC patients (hazard ratio [HR]: 1.98, 95% CI 1.17–3.35, P = 0.011; Table 2). Likewise, advanced T-stage (HR: 3.17, 95% CI 1.34–7.53, P = 0.009), presence of vessel involvement (HR: 7.2, 95% CI 1.3–39.9, P = 0.024), LNM (HR: 4.76, 95% CI 1.42–15.9, P = 0.011), and hyper-editing status of *AZIN1* RNA (HR: 4.55, 95% CI 2.12–9.78, P = 0.0001) emerged as independent prognostic factors for DFS in GC patients (Table 2).

**Table 2:** Univariate and multivariate analysis of various prognostic factors in GC patients

Variables	Univariate			Multivariate		
	HR	95% CI	*P value*	HR	95% CI	*P value*
Overall survival (OS)						
Gender (male)	0.81	0.45–1.45	0.48	0.41	0.94–2.74	0.01*
Age (> 69 year-old)^a^	1.26	0.77–2.06	0.36	1.61	0.21–0.81	0.08
Histological type (Intestinal type)	0.93	0.57–1.51	0.76	0.96	0.57–1.64	0.89
T classification (pT3/4)	3.63	1.92–6.84	0.0001*	2.34	1.2–4.58	0.013*
Vessel involvement (present)	4.12	1.65–10.3	0.002*	5.18	1.33–20.1	0.018*
Lymphatic vessel involvement (present)	2.08	0.65–6.62	0.22	0.14	0.02–0.82	0.029*
Lymph node metastasis (present)	3.86	1.9–7.84	0.0002*	2.86	1.26–6.47	0.012*
Distant metastasis (present)	4.15	2.53–6.81	< 0.0001*	2.56	1.46–4.49	0.001*
*AZIN1* RNA hyper-editing^b^	1.89	1.15–3.08	0.011*	1.98	1.17–3.35	0.011*
Disease free survival (DFS)						
Gender (male)	1.45	0.56–3.75	0.44	0.48	0.15–1.53	0.21
Age (> 69 year-old)^a^	1.18	0.61–2.27	0.62	0.96	0.48–1.94	0.92
Histological type (Intestinal type)	1.38	0.72–2.66	0.33	1.31	0.65–2.65	0.45
T classification (pT3/4)	4.38	1.99–9.64	0.0002*	3.17	1.34–7.53	0.009*
Vessel involvement (present)	3.08	1.19–7.96	0.02*	7.2	1.3–39.9	0.024*
Lymphatic vessel involvement (present)	1.8	0.55–5.88	0.33	0.12	0.01–1.06	0.06
Lymph node metastasis (present)	7.21	2.54–20.5	0.0002*	4.76	1.42–15.9	0.011*
*AZIN1* RNA hyper-editing^b^	5.18	2.63–10.2	< 0.0001*	4.55	2.12–9.78	0.0001*

Variables	Univariate			Multivariate		
	OR	95% CI	*P value*	OR	95% CI	*P value*
Lymph node metastasis (LNM)						
Gender (male)	1.41	0.6–3.3	0.43	1.45	0.55–3.83	0.45
Age (> 69 year-old)^a^	1.7	0.82–3.52	0.16	1.57	0.68–3.65	0.29
Histological type (Intestinal type)	1.17	0.57–2.41	0.66	1.3	0.55–3.08	0.55
T classification (pT3/4)	2.98	1.41–6.29	0.004*	2.15	0.9–5.15	0.09
Vessel involvement (present)	6.89	2.83–16.8	< 0.0001*	5.2	1.66–16.3	0.005*
Lymphatic vessel involvement (present)	8.29	2.12–32.5	0.002*	2.31	0.45–11.8	0.31
*AZIN1* RNA hyper-editing^b^	2.52	1.14–5.56	0.023*	3.03	1.19–7.71	0.02*

*HR* hazard ratio,* OR* odds ratio

* *P *< 0.05

^a^The median age at surgery is 69 years in GC patients

^b^Cut-off thresholds of *AZIN1* RNA editing are determined by ROC analysis with Youden’s index for each outcome in GC patients

### Correlation between editing status of *AZIN1* and ADAR1 expression in GC tissues

We evaluated the relationship between *AZIN1* RNA editing status and ADAR1 expression in GC patients, and showed that *AZIN1* RNA editing was positively correlated with ADAR1 expression in this cohort (P < 0.0001, r = 0.45; Fig. 2c).

### *AZIN1* editing status as an independent risk marker for lymph node metastasis in GC patients

Increased *AZIN1* editing emerged as an independent prognostic factor for OS and DFS, and significantly correlated with the presence of LNM in GC patients. Based on these findings, we next carried out multivariate logistic analysis to determine its clinical significance as a risk marker of LNM in GC patients (Table 2). Intriguingly, we observed that hyper-editing of *AZIN1* RNA was observed to be an independent risk factor associated with LNM; highlighting its potential clinical relevance as an identification of high-risk populations harboring LNM in GC patients.

## Discussion

Emerging evidence indicates that epigenetic alterations encompassing RNA editing may play a central role in post-transcriptional gene regulation, and might control the expression of various cancer-related genes. However, the role of RNA-editing and the expression patterns of its regulatory enzymes, as well as their clinical significance in patients with gastric cancer (GC) remains unclear. Our study provides first evidence supporting a potential clinical impact of dysregulated RNA editing and its regulatory enzymes in GC. First, we observed that *AZIN1* RNA editing levels were significantly higher in GC tissues compared to matched normal mucosa. Second, we noted that the expression of AZIN1-regulatory enzyme, ADAR1, was also up-regulated in GC tissues, and significantly correlated with increased RNA editing in this malignancy. Third, increased *AZIN1* RNA editing and ADAR1 over-expression in GC tissues significantly correlated with key clinico-pathological factors for disease progression, including advanced tumor depth, the presence of lymph node and distant metastases, and higher TNM stages in GC patients. Fourth, both *AZIN1* RNA hyper-editing and elevated ADAR1 expression significantly correlated with poor overall survival (OS), with significant associations between *AZIN1* editing and poor disease free survival (DFS). Fifth, multivariate Cox regression analysis revealed that increased *AZIN1* editing emerged as an independent prognostic factor for both OS and DFS in GC patients. Finally, our data revealed that higher levels of *AZIN1* RNA editing were an independent risk factor for lymph node metastasis (LNM) in patients with GC, highlighting its biomarker potential in the identification of high-risk patients that may experience tumor recurrence post-surgical treatments.

Cancer cells acquire specific characteristics, including escape of cell cycle checkpoint controls, continuous proliferation, invasion, and metastasis. Over the past decades, data suggest that alterations in DNA sequences of the key tumor suppressors and oncogenes may influence the progression of tumorigenesis through functional control of the proteins encoded by these genes [22]. Although these genetic factors are undeniably important, recent years have witnessed an increased emphasis on the contributions of epigenetic alterations mediating cancer pathogenesis, as well as their role as disease biomarkers in various cancer types. Well-studied epigenetic alterations to date include aberrant DNA methylation, and dysregulated expression of noncoding RNAs (e.g. microRNAs, long non-coding RNAs), and various histone modifications. In this context, RNA editing has recently emerged as one of the most recent discoveries, and the most common type of RNA editing in humans is A-to-I conversion catalyzed by ADAR enzymes [1, 23, 24]. RNA editing is consequential, since it directly impacts protein coding sequences, through aberrant transcript splicing, instability, and dysregulated expression levels [25], vindicating the paradigm that site-specific dysregulation of RNA editing may play a pivotal role in cancer development [26–29]. Chen and colleagues performed the first systematic and comprehensive analysis using a sequencing-based approach [6], and demonstrated that *AZIN1* RNA was specifically enhanced in HCC tissues, and that significantly correlated with disease progression in HCC patients. This group recently expanded their research to other cancers within the upper gastrointestinal tract, and successfully demonstrated that reciprocal changes in ADAR1 and ADAR2 coordinated cancer pathogenesis via hypo-editing of the podocalyxin-like protein 1 gene (*PODXL*) [30]. However, the clinical significance of *AZIN1* RNA editing and expression pattern of ADAR1 in GC patients remains unclear; which was the very basis of undertaking the present study. A key finding of our current study was our observation for the increased RNA editing levels of *AZIN1*, as well as dysregulated expression of its regulatory enzyme, ADAR1, in GC tissues vis-à-vis normal gastric mucosa. To date, overexpression of ADAR1 and increased *AZIN1* RNA editing in cancer tissues has been demonstrated in various other types of cancers, including HCC, non-small-cell lung [31], esophageal [9], and colorectal cancer [17]. In line with these evidences, our study for the first time revealed that such dysregulated pattern in GC tissues, and suggested that altered ADAR1 expression and *AZIN1* RNA editing is an important phenomenon in this malignancy as well. Furthermore, enhanced *AZIN1* RNA editing and over-expression of ADAR1, significantly correlated with poor OS and *AZIN1* RNA editing also significantly correlated with poor DFS. Hyper-edited levels of *AZIN1* RNA were an independent prognostic factor for both OS and DFS in GC patients. Taken together, our data for the first time, suggest that measurement of *AZIN1* RNA editing status could be a promising prognostic biomarker for tumor recurrence and survival in patients with GC.

Our study also demonstrated an intimate correlation between *AZIN1* RNA editing levels and lymph node metastasis in GC patients. The presence of regional LNM mainly affects disease recurrence and prognosis in GC patients following curative resection. While recent neoadjuvant therapies may offer treatment options for GC patients with LNM [32, 33], and endoscopic techniques for primary tumor resection, (e.g. endoscopic resection and laparoscopic-assisted gastrectomy) may be used to treat early GC patients without LNM [34], neither of these approaches are robust at identification of high-risk GC patients. Therefore, an accurate risk assessment of which patients may be truly LNM-positive might reduce overly invasive surgeries and improve the prognosis in GC patients. Interestingly, the current study clearly demonstrated that hyper-editing status of *AZIN1* RNA in primary tumor tissues significantly correlated with the presence of LNM, while logistic regression analysis identified it to be an independent risk factor for identifying LNM in GC patients. These findings suggest that the assessment of *AZIN1* RNA editing levels in pre-surgical biopsy specimens might potentially help in the identification of high-risk GC patients with lymph node metastasis.

We would like to acknowledge several potential limitations of our study. First, we focused on *AZIN1* as a most representative RNA editing site in this study, and emerging evidence suggests that there may be potentially other oncogenic RNA editing sites in other genes, which remain an area of active investigation. Therefore, further studies including a broader, unbiased comprehensive analysis may potentially identify additional RNA editing-based markers to assess the risk for oncological outcomes with a higher sensitivity and specificity in GC patients in future. Second, although we have successfully demonstrated our novel findings using a relative-large cohort GC specimens, our cohort was still somewhat smaller, retrospective in nature, and lacked an external independent validation cohort GC specimens. In addition, the clinical materials analyzed in this study were solely from Asian patients, and analytical method for *AZIN1* RNA editing level maybe influenced by several cofounding factors. To overcome these hurdles, larger prospective, multi-institutional studies using same analytical method may be needed to further clarify the prognostic potential of *AZIN1* RNA editing and ADAR1 expression for GC patients.

## Conclusions

In conclusion, this study provides first evidence for the clinical significance of dysregulated *AZIN1* RNA editing and altered expression of its regulatory enzyme ADAR1 in GC. These findings highlight the overarching importance of even small changes in ADAR regulatory cascade, and suggest that *AZIN1* RNA editing may serve as a potential prognostic biomarker in GC patients, and may assist in clinical decision-making for deciding pre-operative treatment strategies in GC patients, as we usher into the era of precision medicine for cancer patients.

## Additional file

**Additional file 1: Table S1.** Primers used for quantitative real time PCR analysis.

## References

[1] Nishikura K (2010). Functions and regulation of RNA editing by ADAR deaminases. Annu Rev Biochem.

[2] Savva YA, Rieder LE, Reenan RA (2012). The ADAR protein family. Genome Biol.

[3] Slotkin W, Nishikura K (2013). Adenosine-to-inosine RNA editing and human disease. Genome Med.

[4] Farajollahi S, Maas S (2010). Molecular diversity through RNA editing: a balancing act. Trends Genet TIG.

[5] Qi L, Chan TH, Tenen DG, Chen L (2014). RNA editome imbalance in hepatocellular carcinoma. Can Res.

[6] Chen L, Li Y, Lin CH, Chan TH, Chow RK, Song Y, Liu M, Yuan YF, Fu L, Kong KL, Qi L, Li Y (2013). Recoding RNA editing of AZIN1 predisposes to hepatocellular carcinoma. Nat Med.

[7] Silva TM, Cirenajwis H, Wallace HM, Oredsson S, Persson L (2015). A role for antizyme inhibitor in cell proliferation. Amino Acids.

[8] Olsen RR, Chung I, Zetter BR (2012). Knockdown of antizyme inhibitor decreases prostate tumor growth in vivo. Amino Acids.

[9] Qin YR, Qiao JJ, Chan TH, Zhu YH, Li FF, Liu H, Fei J, Li Y, Guan XY, Chen L (2014). Adenosine-to-inosine RNA editing mediated by ADARs in esophageal squamous cell carcinoma. Can Res.

[10] Siegel R, Naishadham D, Jemal A (2013). Cancer statistics, 2013. CA Cancer J Clin.

[11] Shimada H, Noie T, Ohashi M, Oba K, Takahashi Y (2014). Clinical significance of serum tumor markers for gastric cancer: a systematic review of literature by the Task Force of the Japanese Gastric Cancer Association. Gastric Cancer.

[12] Malfertheiner P, Megraud F, O’Morain CA, Gisbert JP, Kuipers EJ, Axon AT, Bazzoli F, Gasbarrini A, Atherton J, Graham DY, Hunt R, Moayyedi P (2017). Management of Helicobacter pylori infection-the Maastricht V/Florence Consensus Report. Gut.

[13] Toiyama Y, Takahashi M, Hur K, Nagasaka T, Tanaka K, Inoue Y, Kusunoki M, Boland CR, Goel A (2013). Serum miR-21 as a diagnostic and prognostic biomarker in colorectal cancer. J Natl Cancer Inst.

[14] Han TS, Hur K, Xu G, Choi B, Okugawa Y, Toiyama Y, Oshima H, Oshima M, Lee HJ, Kim VN, Chang AN, Goel A (2015). MicroRNA-29c mediates initiation of gastric carcinogenesis by directly targeting ITGB1. Gut.

[15] Okugawa Y, Toiyama Y, Hur K, Toden S, Saigusa S, Tanaka K, Inoue Y, Mohri Y, Kusunoki M, Boland CR, Goel A (2014). Metastasis-associated long non-coding RNA drives gastric cancer development and promotes peritoneal metastasis. Carcinogenesis.

[16] Okugawa Y, Toiyama Y, Toden S, Mitoma H, Nagasaka T, Tanaka K, Inoue Y, Kusunoki M, Boland CR, Goel A (2017). Clinical significance of SNORA42 as an oncogene and a prognostic biomarker in colorectal cancer. Gut.

[17] Shigeyasu K, Okugawa Y, Toden S, Miyoshi J, Toiyama Y, Nagasaka T, Takahashi N, Kusunoki M, Takayama T, Yamada Y, Fujiwara T, Chen L (2018). AZIN1 RNA editing confers cancer stemness and enhances oncogenic potential in colorectal cancer. JCI Insight.

[18] Japanese-Gastric-Cancer-Association. Japanese Classification of Gastric Carcinoma 2010;14:10–25. 10.1007/s101209800016 11957040

[19] Crews LA, Jiang Q, Zipeto MA, Lazzari E, Court AC, Ali S, Barrett CL, Frazer KA, Jamieson CH (2015). An RNA editing fingerprint of cancer stem cell reprogramming. J Transl Med.

[20] Jiang Q, Crews LA, Barrett CL, Chun HJ, Court AC, Isquith JM, Zipeto MA, Goff DJ, Minden M, Sadarangani A, Rusert JM, Dao KH (2013). ADAR1 promotes malignant progenitor reprogramming in chronic myeloid leukemia. Proc Natl Acad Sci USA.

[21] Han L, Diao L, Yu S, Xu X, Li J, Zhang R, Yang Y, Werner HMJ, Eterovic AK, Yuan Y, Li J, Nair N (2015). The genomic landscape and clinical relevance of A-to-I RNA editing in human cancers. Cancer Cell.

[22] Okugawa Y, Grady WM, Goel A (2015). Epigenetic alterations in colorectal cancer: emerging biomarkers. Gastroenterology.

[23] Bass BL (2002). RNA editing by adenosine deaminases that act on RNA. Annu Rev Biochem.

[24] Levanon EY, Eisenberg E, Yelin R, Nemzer S, Hallegger M, Shemesh R, Fligelman ZY, Shoshan A, Pollock SR, Sztybel D, Olshansky M, Rechavi G (2004). Systematic identification of abundant A-to-I editing sites in the human transcriptome. Nat Biotechnol.

[25] Wang IX, So E, Devlin JL, Zhao Y, Wu M, Cheung VG (2013). ADAR regulates RNA editing, transcript stability, and gene expression. Cell Rep.

[26] Nemlich Y, Greenberg E, Ortenberg R, Besser MJ, Barshack I, Jacob-Hirsch J, Jacoby E, Eyal E, Rivkin L, Prieto VG, Chakravarti N, Duncan LM (2013). MicroRNA-mediated loss of ADAR1 in metastatic melanoma promotes tumor growth. J Clin Investig.

[27] Galeano F, Rossetti C, Tomaselli S, Cifaldi L, Lezzerini M, Pezzullo M, Boldrini R, Massimi L, Di Rocco CM, Locatelli F, Gallo A (2013). ADAR2-editing activity inhibits glioblastoma growth through the modulation of the CDC14B/Skp2/p21/p27 axis. Oncogene.

[28] Gallo A, Galardi S (2008). A-to-I RNA editing and cancer: from pathology to basic science. RNA Biol.

[29] Paz N, Levanon EY, Amariglio N, Heimberger AB, Ram Z, Constantini S, Barbash ZS, Adamsky K, Safran M, Hirschberg A, Krupsky M, Ben-Dov I (2007). Altered adenosine-to-inosine RNA editing in human cancer. Genome Res.

[30] Chan TH, Qamra A, Tan KT, Guo J, Yang H, Qi L, Lin JS, Ng VH, Song Y, Hong H, Tay ST, Liu Y (2016). ADAR-mediated RNA editing predicts progression and prognosis of gastric cancer. Gastroenterology.

[31] Hu X, Chen J, Shi X, Feng F, Lau KW, Chen Y, Chen Y, Jiang L, Cui F, Zhang Y, Xu X, Li J (2017). RNA editing of AZIN1 induces the malignant progression of non-small-cell lung cancers. Tumour Biol.

[32] Schwarz RE (2015). Current status of management of malignant disease: current management of gastric cancer. J Gastrointest Surg.

[33] Newton AD, Datta J, Loaiza-Bonilla A, Karakousis GC, Roses RE (2015). Neoadjuvant therapy for gastric cancer: current evidence and future directions. J Gastrointest Oncol.

[34] Yoshii T, Miyagi Y, Nakamura Y, Kobayashi O, Kameda Y, Ohkawa S (2013). Pilot research for the correlation between the expression pattern of E-cadherin-beta-catenin complex and lymph node metastasis in early gastric cancer. Tumori.

