# Supplementary material for: Enhanced AZIN1 RNA editing and overexpression of its regulatory enzyme ADAR1 are important prognostic biomarkers in gastric cancer
Source: J Transl Med. 2018 Dec 18;16:366. doi: 10.1186/s12967-018-1740-z (PMC6299520; doi:10.1186/s12967-018-1740-z)
Supplement: Supplementary file 1 — Additional file 1: Table S1. Primers used for quantitative real time PCR analysis. [file 12967_2018_1740_MOESM1_ESM.docx]

**Clinical significance of *AZIN1* RNA editing and its regulatory enzyme ADAR1 in gastric cancer**

Yoshinaga Okugawa M.D, Ph.D^1,2,*^, Yuji Toiyama M.D, Ph.D, FACS^1^, Kunitoshi Shigeyasu M.D, Ph.D^2^, Akira Yamamoto M.D^1^, Tsunehiko Shigemori M.D^1^, Chengzeng Yin M.D^1^, Takashi Ichikawa M.D^1^, Hiromi Yasuda M.D, Ph.D^1^, Hiroyuki Fujikawa M.D, Ph.D^1^, Shigeyuki Yoshiyama M.D, Ph.D^1^, Junichiro Hiro M.D, Ph.D^1^, Masaki Ohi M.D, Ph.D^1^, Toshimitsu Araki M.D, Ph.D^1^, Masato Kusunoki M.D, Ph.D^1^, and Ajay Goel Ph.D^3^

^1^ Department of Gastrointestinal and Pediatric Surgery, Division of Reparative Medicine, Institute of Life Sciences, Mie University Graduate School of Medicine, Japan

^2^ Department of Gastroenterological Surgery, Okayama University Graduate School of Medicine, Dentistry, and Pharmaceutical Sciences, Okayama, Japan

^3^ Center for Gastrointestinal Research and Center for Translational Genomics and Oncology, Baylor Scott & White Research Institute and Charles A. Sammons Cancer Center, Baylor University Medical Center, Dallas, Texas, USA

**Additional File**

| **Additional File 1:** Primers used for quantitative real time PCR analysis | |  |
| --- | --- | --- |
|  | | |
| **Primer for RNA editing site-specific quantitative PCR (RESSqPCR)** | | |
| Gene | Forward | Reverse |
| Wild *AZIN1* | CATTCAGCTCAGGAAGAAGACATCT | AATACAAGGAAGATGAGCCTCTGTTTAC |
| Edit *AZIN1* | ACTGAATGACATCATGTAATAAATGGCT | GAGCTTGATCAAATTGTGGCAG |
| **Primer for Gene expression quantitative PCR (qPCR)** | | |
| Gene | Forward | Reverse |
| ADAR1 | CCCTTCAGCCACATCCTTC | GCCATCTGCTTTGCCACTT |
| GAPDH | GGAAGGTGAAGGTCGGAGTC | AATGAAGGGGTCATTGATGG |
| ADAR: Adenosine deaminase acting on RNA, GAPDH: glyceraldehyde 3-phosphate dehydrogenase | | |
